# A cross-reactive antibody protects against Ross River virus musculoskeletal disease despite rapid neutralization escape in mice

**DOI:** 10.1371/journal.ppat.1008743

**Published:** 2020-08-06

**Authors:** Julie M. Fox, Ling Huang, Stephen Tahan, Laura A. Powell, James E. Crowe, David Wang, Michael S. Diamond

**Affiliations:** 1 Department of Medicine, Washington University in St. Louis, St. Louis, Missouri, United States of America; 2 MacroGenics, Rockville, Maryland, United States of America; 3 Department of Molecular Microbiology, Washington University in St. Louis, St. Louis, Missouri, United States of America; 4 Department of Pathology, Microbiology and Immunology, Vanderbilt University Medical Center, Nashville, Tennessee, United States of America; 5 Vanderbilt Vaccine Center and Department of Pediatrics, Vanderbilt University Medical Center, Nashville, Tennessee, United States of America; 6 Department of Pathology and Immunology, Washington University in St. Louis, St. Louis, Missouri, United States of America; Emory University, UNITED STATES

## Abstract

Arthritogenic alphaviruses cause debilitating musculoskeletal disease and historically have circulated in distinct regions. With the global spread of chikungunya virus (CHIKV), there now is more geographic overlap, which could result in heterologous immunity affecting natural infection or vaccination. Here, we evaluated the capacity of a cross-reactive anti-CHIKV monoclonal antibody (CHK-265) to protect against disease caused by the distantly related alphavirus, Ross River virus (RRV). Although CHK-265 only moderately neutralizes RRV infection in cell culture, it limited clinical disease in mice independently of Fc effector function activity. Despite this protective phenotype, RRV escaped from CHK-265 neutralization *in vivo*, with resistant variants retaining pathogenic potential. Near the inoculation site, CHK-265 reduced viral burden in a type I interferon signaling-dependent manner and limited immune cell infiltration into musculoskeletal tissue. In a parallel set of experiments, purified human CHIKV immune IgG also weakly neutralized RRV, yet when transferred to mice, resulted in improved clinical outcome during RRV infection despite the emergence of resistant viruses. Overall, this study suggests that weakly cross-neutralizing antibodies can protect against heterologous alphavirus disease, even if neutralization escape occurs, through an early viral control program that tempers inflammation.

## Author summary

The induction of broadly neutralizing antibodies is a goal of many antiviral vaccine programs. In this study, we show that cross-reactive monoclonal and polyclonal antibodies developed after CHIKV infection or immunization with relatively weak cross-neutralizing activity can protect against RRV-induced musculoskeletal disease in mice. Even though RRV rapidly escaped from neutralization, antibody therapy reduced inflammation in musculoskeletal tissues and decreased viral burden near the site of infection in a manner that required type I interferon signaling. These studies in mice show that broadly reactive antibodies with limited neutralizing activity still can confer protection against heterologous alphaviruses.

## Introduction

Alphaviruses are arthropod-transmitted, single-stranded, positive-sense enveloped RNA viruses in the *Togaviridae* family. The arthritogenic alphaviruses, including chikungunya (CHIKV), Ross River (RRV) and Mayaro (MAYV) viruses, cause fever, rash, and debilitating arthritis and musculoskeletal disease, with some symptoms lasting for months to years [1–4]. Historically, these viruses circulated in distinct regions of the world with CHIKV causing periodic outbreaks in Africa and Asia, RRV circulating in Australia and the Pacific Islands, and MAYV causing infections in Central and South America [5]. However, in the mid-2000s, CHIKV caused explosive outbreaks in Africa and Asia [6], and in 2013, CHIKV emerged in the Caribbean and spread to Central and South America causing over 1.7 million cases including locally acquired cases in Florida [7]. Currently, there is no approved vaccine or therapeutic for any arthritogenic alphavirus, although multiple candidate CHIKV vaccines are advancing through clinical trials [8].

The alphavirus genome encodes four non-structural (nsp1 to nsp4) and five structural proteins (capsid, E3, E2, 6K, and E1) from two open reading frames. In an infected cell, p62 (E3-E2) and E1 form heterodimers in the endoplasmic reticulum. E3 is cleaved by furin in the trans-Golgi compartment and then E2-E1 is transported to the plasma membrane where virion morphogenesis and budding occur [9]. The virion displays 240 E2-E1 heterodimers as 80 trimers in a T = 4 quasi-icosahedral symmetry [10, 11], and these proteins mediate virus attachment and internalization in part through interaction with a cognate receptor, Mxra8 [12–14]. The E2 protein contains three immunoglobulin domains: A domain, which is centrally located; B domain, which is located at the tip of the heterodimer; and the C domain, which is proximal to the virion surface. The E1 protein is a type II membrane fusion protein that contains three β-sheet domains (I, II, III). Domain I is located between domains II and III. Domain II is located distal to the center of the trimer, contains the fusion peptide, and is covered by the E2 B domain [10]. The E2 and E1 glycoproteins are the principal antigenic targets of neutralizing antibodies.

Multiple studies have demonstrated antibody-based protection against alphavirus infection in animal models. Passive transfer of CHIKV immune human IgG protects mice from a lethal CHIKV challenge [15]. Potently neutralizing monoclonal antibodies (mAbs) against CHIKV and MAYV reduce viral burden and clinical disease when administered therapeutically [16–23]. CHIKV vaccine candidates induce neutralizing antibody responses in humans and limit viral burden and disease in mice and non-human primates following challenge [8]. In addition to virus-specific protection, some mAbs against CHIKV or MAYV cross-neutralize related alphaviruses, and pre-existing immunity to CHIKV or RRV infection reduces clinical disease following challenge with MAYV or CHIKV, respectively [24–28]. We previously described mAbs against CHIKV that bind the E2 B domain and broadly neutralize multiple arthritogenic alphaviruses with a range of potencies [29]. Despite the potential geographical overlap of endemic alphaviruses and CHIKV, as well as possible deployment of promising CHIKV vaccines, the impact of cross-reactive humoral immunity on heterologous alphavirus infection and disease has not been studied extensively.

Here, we evaluated the efficacy of an anti-CHIKV mAb, CHK-265, to protect *in vivo* against RRV, a virus that it only weakly neutralizes, using an established mouse model of RRV-induced musculoskeletal disease [30]. We show that prophylaxis of CHK-265 reduces RRV-mediated disease independently of FcγR interactions. Although CHK-265 initially reduced viral burden, RRV rapidly escaped from CHK-265 neutralization by selecting for mutations in the E2 B domain. In parallel experiments, we show that cross-reactive CHIKV immune human polyclonal IgG also protects against RRV disease *in vivo* despite the emergence of RRV variants that reduce binding to the CHIKV immune IgG. Notwithstanding this escape, CHK-265 treatment reduced infection at the site of inoculation at very early time points resulting in diminished levels of pro-inflammatory cytokines and chemokines and infiltration of immune cells. Thus, even relatively weakly cross-neutralizing anti-CHIKV antibodies reduce RRV-induced disease by limiting of immune-mediated inflammation in musculoskeletal tissues.

## Results

### Cross-reactive anti-CHIKV mAb reduces RRV-induced disease in an Fc effector function-independent manner

**Fig 1:**
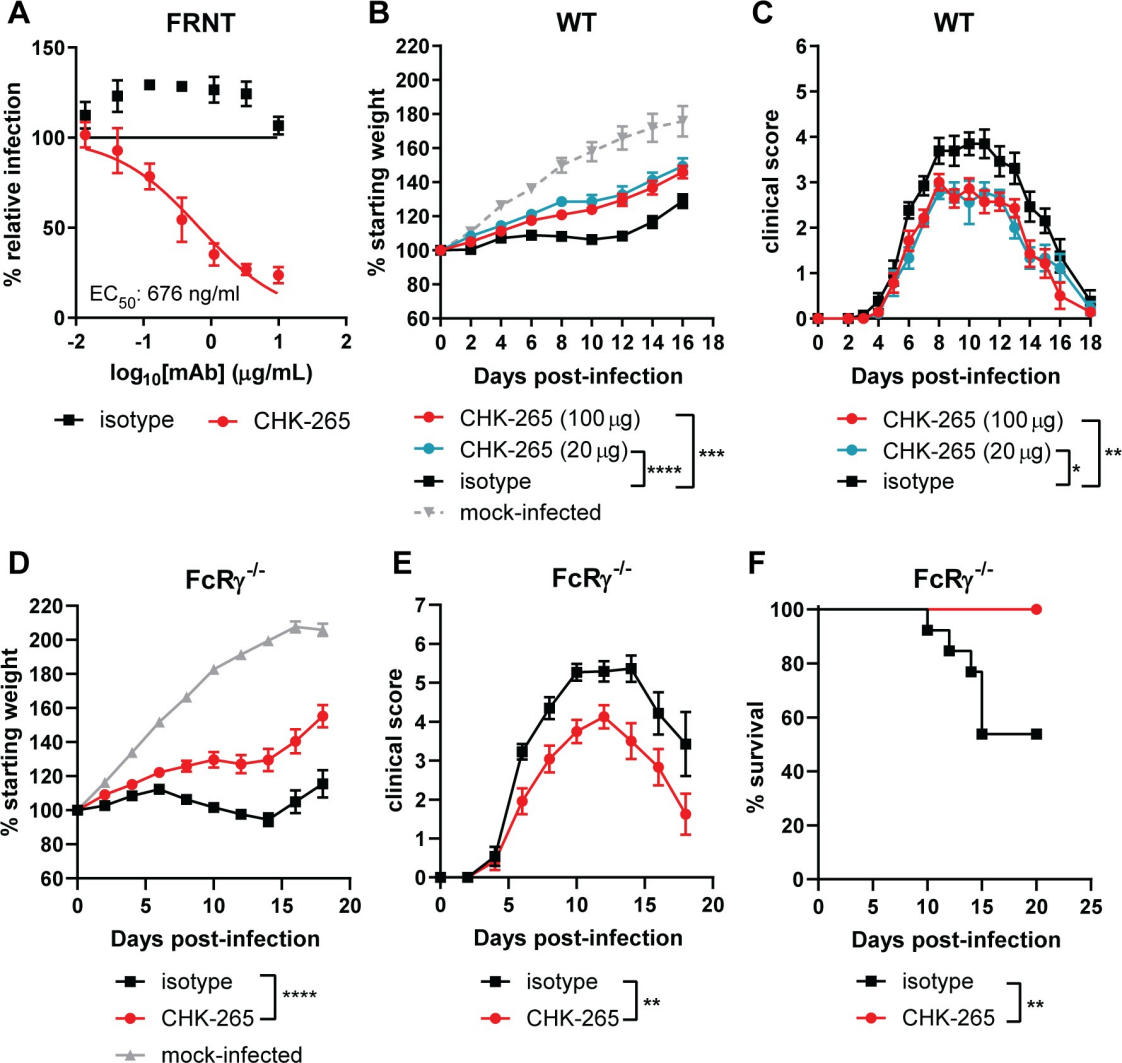
Prophylaxis with CHK-265 reduces RRV disease *in vivo*. (**A**) Neutralization assay. CHK-265 was preincubated with 10^2^ FFU of RRV and added to Vero cells for 20 h. Viral foci were counted and compared to wells with no mAb to determine relative infection. WNV E60 was included as an isotype control. Graph represents the mean ± SD (two experiments performed in duplicate) and are from the same experiments as Fig 2G. (**B-C**) Three-week-old male and female WT C57BL/6 or (**D-F**) FcRγ^-/-^ mice were administered (**B-F**) 100 μg or (**B-C**) 20 μg of CHK-265 or an isotype control mAb one day prior to infection with 10^3^ FFU of RRV. (**B, D**) Weights, (**C, E)** clinical disease, (**F**) survival were recorded [(**B-C**) n = 9–14 per group, three experiments; (**D-F**) n = 12–13 per group, three experiments]. Graphs show mean values ± SEM [(**B-C**): One-way ANOVA of area under the curve (AUC) analysis with a Dunnett’s post-test comparing each group to the isotype control mAb. (**D-E**) Unpaired t-test of AUC analysis: ***P < 0.001, ****P < 0.0001; (**F**) log-rank test: **P < 0.01].

We previously identified anti-CHIKV mAbs that bind to the E2 B domain and have a range of inhibitory activity against heterologous arthritogenic alphaviruses [29]. One anti-CHIKV mAb, CHK-265 (IgG2c), potently neutralizes Mayaro virus (MAYV) infection (EC_50_ of 4 ng/ml) [29] but inhibits RRV less efficiently (**Fig 1A,** EC_50_ of 676 ng/ml). To test whether the moderately neutralizing activity of CHK-265 against RRV protects *in vivo*, we tested it in an established model of musculoskeletal disease in three- to four-week old wild-type (WT) C57BL/6 mice [30]. We administered 20 or 100 μg of CHK-265 or isotype control mAb one day before subcutaneous inoculation in the foot of 10^3^ focus-forming units (FFU) of RRV. A clinical disease score [31] was used, which evaluated hindpaw grip strength, gait, righting reflex, and weight change. Mice receiving either dose of CHK-265 gained more weight than the isotype control mAb-treated mice after RRV infection, although treatment did not restore weight gain to levels in mock-infected mice (**Fig 1B**). Mice administered either dose of CHK-265 also had an improved clinical score compared to isotype control mAb treatment (**Fig 1C**).

Therapeutic administration of some anti-CHIKV mAbs protect against CHIKV-induced joint swelling in a manner that requires Fc-FcγR interactions [32]. To determine if CHK-265 reduced RRV-induced clinical disease through a similar mechanism, we repeated studies in mice lacking the common γ signaling chain of activating FcγRs (FcRγ^-/-^). Notably, CHK-265 treatment still resulted in increased weight gain, improved clinical score, and increased survival in FcRγ^-/-^ mice compared to the isotype control mAb (**Fig 1D–1F**). Thus, CHK-265 mediates disease reduction independently of Fc-FcγRs interactions, possibly through direct neutralization of RRV.

### CHK-265 mAb shifts the kinetics of RRV infection and dissemination

**Fig 2:**
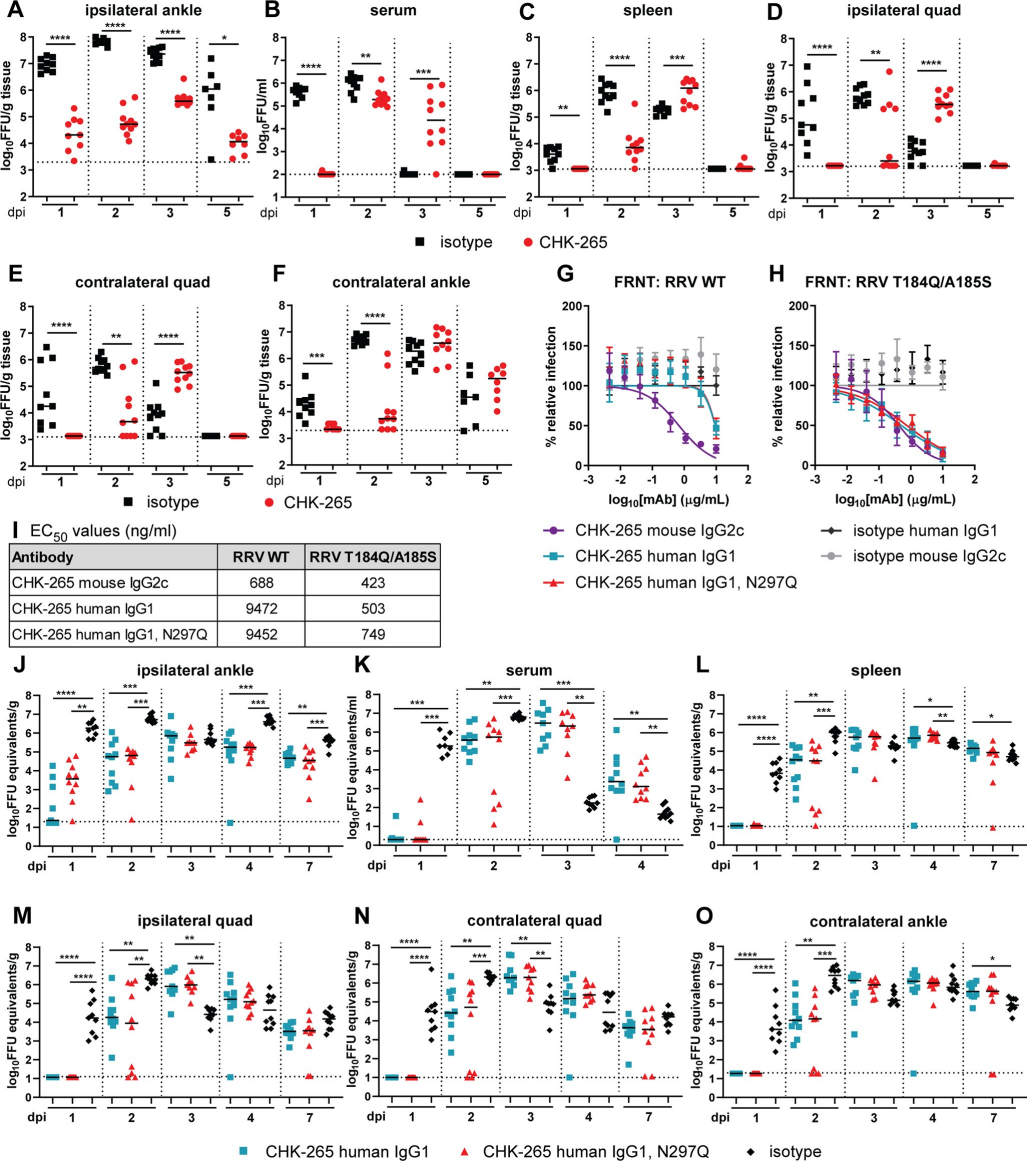
CHK-265 shifts the kinetics of RRV infection and dissemination independently of Fc effector interactions. Four-week-old male and female WT C57BL/6 mice were administered 100 μg of CHK-265 one day before infection with 10^3^ FFU of RRV. (**A**) Ipsilateral ankle, (**B**) serum, (**C**) spleen, (**D**) ipsilateral and (**E**) contralateral quadriceps muscles (quad), and (**F**) contralateral ankle were harvested at indicated time points, and viral titers were determined by focus-forming assay [FFA (n = 7–10 per group; two experiments)]. Bars indicate median values (Mann-Whitney test at each time point for each tissue; *P < 0.05, **P < 0.01, ***P < 0.001, ****P < 0.0001). (**G**-**H**) Neutralization assays. mAbs (CHK-265 mouse IgG2c, CHK-265 human IgG1, or CHK-265 human IgG1 N297Q) were preincubated with 10^2^ FFU of (**G**) RRV WT or (**H**) RRV T184Q/A185S and then added to Vero cells for 20 h. Viral foci were counted and compared to wells without mAbs to determine relative infection. WNV E16 human IgG1 and WNV E60 mouse IgG2c were included as isotype control mAbs. Graphs show mean values ± SD (three experiments performed in duplicate). (**I**) Non-linear regression constraining the top and bottom to 100 and 0, respectively, was used to determine the EC_50_ values of each mAb for each virus. Mean EC_50_ values are shown (three experiments). (**J**-**O**) Four-week-old male and female WT C57BL/6 mice were treated with 50 μg of CHK-265 human IgG1 or CHK-265 human IgG1 N297Q one day prior to inoculation with 10^3^ FFU of RRV T184Q/A185S. (**J**) Ipsilateral ankle, (**K**) serum, (**L**) spleen, (**M**) ipsilateral and (**N**) contralateral quadriceps muscles (quad), and (**O**) contralateral ankle were harvested at indicated time points, and viral titers were determined by qRT-PCR (n = 9–10 per group; two experiments). Bars indicate median values (Kruskal-Wallis with Dunn’s post-test at each time point for each tissue; *P < 0.05, **P < 0.01, ***P < 0.001, ****P < 0.0001).

To begin to determine how CHK-265 reduced clinical disease, we measured viral burden in tissues over the course of infection. We administered 100 μg of CHK-265 one day prior to inoculation of mice with RRV, and evaluated levels of infectious virus in the serum, spleen, ipsilateral and contralateral quadriceps muscles, and ipsilateral and contralateral ankles at 1, 2, 3, or 5 dpi. At 1 and 2 dpi, CHK-265 treatment reduced RRV levels in the ipsilateral ankle and prevented dissemination to other tissues (**Fig 2A–2F**). However, at 2 dpi, CHK-265 treated mice showed increased viremia (**Fig 2B**) and by 3 dpi, paradoxically higher viral burden was observed in the serum, ipsilateral and contralateral quadriceps muscles, and the contralateral ankle in mice treated with CHK-265-compared to isotype control mAb (**Fig 2C–2F**). The viral load in mice treated with CHK-265 in most tissues at 3 dpi was comparable to that observed with isotype mAb treatment at 2 dpi, suggesting a delay in the infection kinetics. The ipsilateral ankle was the only tissue that showed consistently reduced RRV titers after CHK-265 treatment throughout the time course (**Fig 2A**).

To determine if Fc effector functions of antibody contributed to the shift in viral kinetics and dissemination, we generated chimeric wild-type and aglycosyl N297Q variants of CHK-265 with the human IgG1 constant region. The N297Q mutation eliminates an N-linked glycosylation site, which reduces interactions with FcγRs and C1q [33]. Unexpectedly, the recombinant, chimeric variants of CHK-265 showed reduced neutralizing potency of RRV in cell culture compared to the parental mouse form of CHK-265 (**Fig 2G and 2I;** EC_50_: mouse, 688 ng/ml; chimeric human WT, 9472 ng/ml; and chimeric human N297Q, 9452 ng/ml) [34]. To overcome this limitation, which might be due to allosteric effects of the Fc region on antigen binding [34], we used a previously described RRV mutant (E2-T184Q/A185S), in which two residues in the B domain of E2 from CHIKV were introduced into the infectious cDNA clone of RRV and resulted in enhanced neutralization [29]. Indeed, the chimeric human IgG1 variants of CHK-265 mAb neutralized RRV T184Q/A185S to levels that were equivalent to the parental RRV by mouse CHK-265 mAb (**Fig 2G–2I;** EC_50_: mouse, 423 ng/ml; chimeric human WT, 503 ng/ml; and chimeric human N297Q, 749 ng/ml). Based on these data, we used the E2-T184Q/A185S mutant of RRV for infection experiments *in vivo* with WT and N297Q chimeric human IgG1 forms of CHK-265. Mice received 50 μg of chimeric human CHK-265 (WT or N297Q IgG1) or an isotype control mAb one day prior to subcutaneous inoculation with RRV T184Q/A185S. Tissues were collected 1, 2, 3, 4, or 7 dpi and analyzed for viral burden by qRT-PCR. We used qRT-PCR rather than an infectious virus assay, as it provides a longer window of analysis to see the impact of Fc effector functions on viral clearance [32]. Similar to experiments with the mouse CHK-265 and the parental RRV, the serum, spleen, and ipsilateral and contralateral quadriceps muscles and ankle had minimal detectable viral RNA at 1 dpi, and the ipsilateral ankle had a lower level of viral RNA than the isotype mAb treated mice (**Fig 2J–2O**). By 2 dpi, mice treated with either chimeric human CHK-265 (WT or N297Q) sustained RRV infection in all tissues evaluated. The viral burden present at 2 dpi in mice treated with chimeric human CHK-265 (WT or N297Q) was similar to levels present in isotype mAb-treated mice at 1 dpi. This pattern also was observed at several later days in some of the tissues (*e*.*g*., serum, spleen, and quadriceps muscle), with chimeric human CHK-265 (WT or N297Q) treated mice having higher titers than isotype mAb treated mice. However, and as observed with mouse CHK-265 (**Fig 2A**), the ipsilateral ankle of chimeric human CHK-265 (WT or N297Q) treated mice showed less viral infection than the isotype mAb control for the majority of the time course (**Fig 2J**). Collectively, these results corroborate that the shift in viral kinetics and dissemination is independent of Fc-effector functions. To confirm that this phenotype was not specific to the chimeric human mAbs in mice or the mutant RRV, we repeated the studies using mouse CHK-265 mAb in FcRγ^-/-^ or C1q^*-/-*^ and analyzed viral load at 3 dpi by focus-forming assay (FFA). Treatment of FcRγ^-/-^ or C1q^*-/-*^ animals with mouse CHK-265 mAb resulted in increased RRV titers at 3 dpi in all tissues except the ipsilateral ankle compared to the isotype mAb treated mice (**S1 Fig**). Collectively, these results establish that Fc effector functions (via FcγR or C1q interactions) do not contribute to the shift in viral kinetics associated with CHK-265 mAb treatment.

### A RRV escape mutant evades CHK-265 neutralization and replicates efficiently *in vivo*

**Fig 3:**
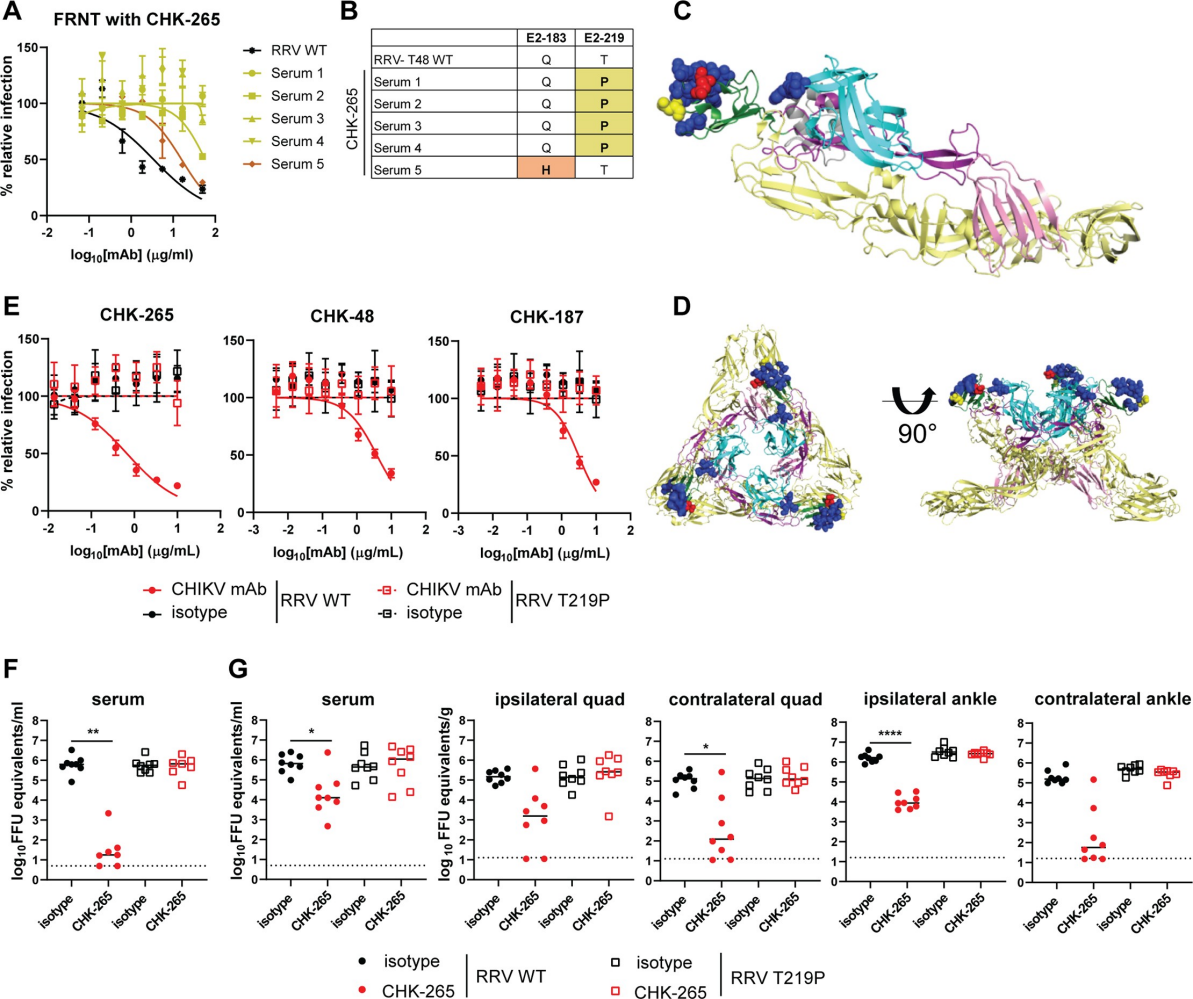
RRV escapes from neutralization by CHK-265. (**A**) Neutralization assay. CHK-265 was preincubated with 10^2^ FFU of RRV derived from serum collected from CHK-265-treated mice at 3 dpi or parental RRV (RRV WT) and then added to Vero cells for 20 h. Viral foci were counted and compared to wells without mAb to determine relative infection. (**B**) Viral RNA was isolated from serum or from RRV WT stock and sequenced. Mutations are highlighted and indicated in bold. (**C**) Critical residues for CHK-265 binding determined by cryo-EM are shown as blue spheres, and identified mutations are shown as red (E2-183) or yellow (E2-219) spheres on the CHIKV p62-E1 structure using PyMOL (PDB 3N42): E1 in yellow, E3 in gray, E2-A in cyan, E2-B in dark green, E2-arch in purple, and E2-C in pink. (**D**) The residues highlighted in (**C**) modeled on the CHIKV trimeric spike using PyMOL (PBD 2XFB). (**E**) Neutralization assay. CHK-265, CHK-48, and CHK-187 were preincubated with 10^2^ FFU of RRV WT or RRV E2-T219P and then added to Vero cells for 20 h. Viral foci were counted and compared to wells without mAb to determine relative infection. WNV E60 was included as an isotype control. Graphs show mean ± SD (two experiments performed in duplicate). (**F-G**) Four-week-old male and female WT C57BL/6 mice were treated with 100 μg of CHK-265 one day prior to inoculation with 10^3^ FFU of RRV WT or RRV E2-T219P. Serum was collected (**F**) 1 or (**G**) 2 dpi, and (**G**) spleen, ipsilateral and contralateral quadriceps muscles (quad), and ipsilateral and contralateral ankles were harvested at 2 dpi. Viral titers were determined by qRT-PCR (n = 7–8 per group; two experiments). Bars indicate median values (Kruskal-Wallis with Dunn’s post-test; *P < 0.05, **P < 0.01, ****P < 0.0001).

To determine whether the shift in viral kinetics was due to neutralization escape of RRV from CHK-265, we evaluated virus derived from the serum of mice treated with CHK-265 at 3 dpi. Whereas CHK-265 neutralized the parental RRV, all of the serum-derived virus samples tested showed shifts in or loss of neutralization by CHK-265 (**Fig 3A**). Four of the five viruses isolated from serum had E2-T219P mutations and one had an E2-Q183H mutation (**Fig 3B**). Both mutations are located within the B domain of the E2 protein proximal to, or within, the binding footprint of CHK-265, which was identified previously using alanine scanning mutagenesis and cryo-electron microscopy (cryoEM) [[29] and **Fig 3C and 3D**, *yellow and red spheres*]. Since escape variants emerged within 3 days of RRV challenge, we sequenced the RRV stock used for inoculation using a deep sequencing technique (Illumina MiSeq) to determine if these were pre-existing variants. The E2-T219P variant was present at a 3.5% frequency, whereas the Q183H variant was absent (**S1 Table**). Additional amino acid variants within the CHK-265 footprint were identified in the RRV stock used for inoculation (**S2 Table**).

To confirm the significance of the E2-T219P mutation, we generated the variant RRV recombinantly using an infectious cDNA clone [35]. CHK-265 and two other cross-neutralizing anti-CHIKV mAbs (CHK-48 and CHK-187) failed to neutralize recombinant RRV E2-T219P in cell culture (**Fig 3E**). We next evaluated if the RRV E2-T219P variant replicated efficiently *in vivo*. If attenuated, this effect of the mutation could explain the reduced clinical disease with CHK-265 mAb treatment. We inoculated RRV WT or E2-T219P virus in the foot, and viral burden was assessed at 1 dpi in serum and 2 dpi in tissues. We included a cohort of mice treated with CHK-265 in combination with E2-T219P to prevent reversion. At 1 and 2 dpi, as expected, CHK-265 treatment resulted in reduced RRV WT viremia. In comparison, there was no difference in viremia at 1 or 2 dpi between RRV WT infected mice treated with isotype control mAb and RRV E2-T219P-infected mice treated with CHK-265 or isotype mAb (**Fig 3F and 3G**). Moreover, mice infected with RRV E2-T219P and treated or not with CHK-265 also had equivalent viral burden at 2 dpi compared to RRV WT-infected mice treated with an isotype control mAb. In contrast, mice treated with CHK-265 and infected with RRV WT had reduced viral titers in musculoskeletal tissues (**Fig 3G**). To confirm that the E2-T219P virus did not revert to a WT sequence, the E2 and E1 genes from viral RNA isolated from serum were analyzed by Sanger sequencing. Mice infected with RRV E2-T219P with or without CHK-265 treatment maintained the mutation without secondary site changes. Virus sequences from mice infected with RRV WT and treated with the isotype control mAb had no mutations, whereas virus sequences from the mice treated with CHK-265 and infected with RRV WT had mutations in the B domain of E2 proximal to the site of T219. Collectively, these results establish that the escape variant generated under CHK-265 mAb selection pressure replicated efficiently *in vivo*. Thus, CHK-265 likely reduces RRV-induced clinical disease through a more complex mechanism than simple reduction in replication.

### Treatment of animals with CHK-265 reduces RRV infection near the site of inoculation through type I IFN signaling

**Fig 4:**
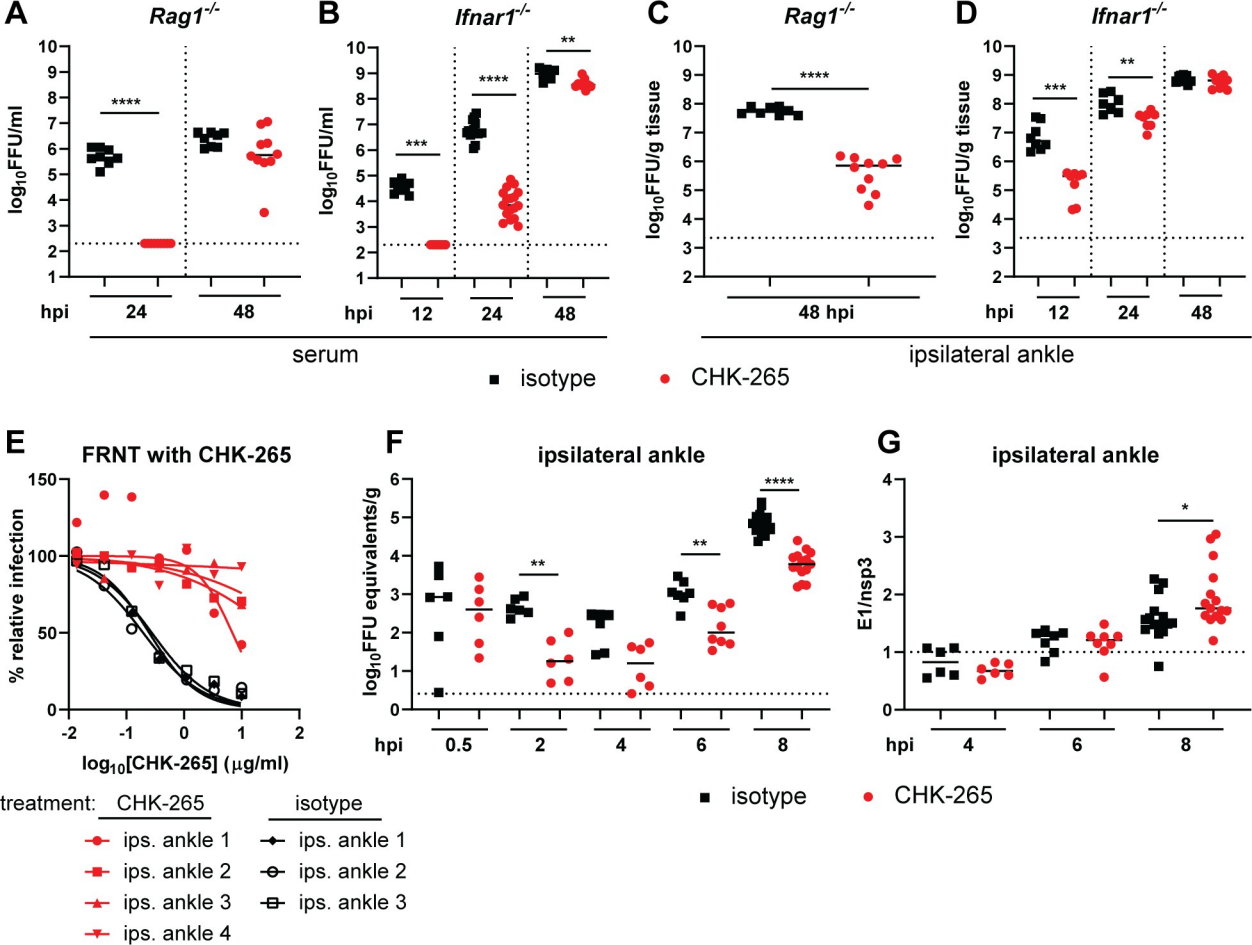
The reduced RRV infection by CHK-265 at the site of inoculation requires type I IFN signaling. Four-week-old male and female *Rag1*^*-/-*^ (**A**, **C**), *Ifnar1*^*-/-*^ (**B**, **D-E**), or WT (**F, G**) C57BL/6 mice were administered 100 μg of CHK-265 prior to infection with 10^3^ FFU of RRV. (**A**-**B**) Serum and (**C**-**D**) ipsilateral ankles were harvested at indicated time points and titered by FFA. Bars indicate median values [(**A**, **C**) n = 8–10 per group; three experiments; (**B**, **D**) n = 7–17 per group; two experiments; Mann-Whitney test; **P < 0.01, ***P < 0.001, ****P < 0.0001]. (**E**) Neutralization assay. CHK-265 was preincubated with 10^2^ FFU of RRV derived from the ipsilateral ankle collected from CHK-265 or isotype-treated *Ifnar1*^*-/-*^ mice at 2 dpi and then added to Vero cells for 20 h. Viral foci were counted and compared to wells without mAb to determine relative infection. (**F**-**G**) Ipsilateral ankles were harvested at indicated time points and (**F**) viral RNA was titered by qRT-PCR for the nsp3 gene. (**G**) RRV E1 and nsp3 genes were quantified by qRT-PCR and the ratio of structural (E1)/ non-structural (nsp3) genes were plotted as an indirect measure of active viral infection. (**F-G**) Bars indicate median (n = 6–15 per group; two to five experiments; Mann-Whitney test; *P < 0.05, **P < 0.01, ****P < 0.0001).

Although CHK-265 mAb treatment initially protects against RRV infection, most tissues, with the exception of the ipsilateral ankle, ultimately sustained similar titers as those treated with isotype control mAb, albeit with delayed kinetics (**Fig 2B–2F**). We hypothesized that early local immune events in the foot induced by antigen-antibody complexes prior to RRV neutralization escape might contribute to the durable reduction in infection near the inoculating site and lead to reduced clinical disease. To test whether adaptive or innate immune response contributed to this reduced viral load, mice lacking T and B cells (*Rag1*^*-/-*^) or the type I IFN receptor (*Ifnar1*^*-/-*^) were treated with CHK-265 mAb prior to inoculation with RRV. Consistent with results in WT mice, CHK-265 reduced viremia in *Rag1*^*-/-*^ and *Ifnar1*^*-/-*^ mice for the first 24 hours post-infection (hpi), and by 48 hpi, both *Rag1*^*-/-*^ and *Ifnar1*^*-/-*^ mice had high viremia comparable to that in isotype mAb-treated mice (**Fig 4A and 4B**). In the ipsilateral ankle, CHK-265 reduced the viral load in *Rag1*^*-/-*^ mice at 2 dpi (**Fig 4C**), consistent with the results from WT mice. However, CHK-265 only controlled RRV infection for the first 24 hpi in the ipsilateral ankle of *Ifnar1*^*-/-*^ mice. By 48 hpi, the viral burden was equivalent between CHK-265 and isotype control mAb-treated *Ifnar1*^*-/-*^ mice (**Fig 4D**). This time point was associated with the emergence of RRV neutralization escape variant viruses in the ipsilateral ankle of both *Ifnar1*^*-/-*^ and WT mice (**Figs 4E and S2**). Collectively, these data suggest that in the context of CHK-265 treatment, the control of RRV infection in the ipsilateral ankle is mediated through type I IFN signaling.

To determine how CHK-265 mAb treatment modified the initial infection *in vivo*, we analyzed ipsilateral ankle tissues for viral RNA levels at 0.5, 2, 4, 6, or 8 h after RRV inoculation. At 0.5 hpi, we did not observe a significant difference in viral RNA levels in ankle tissues from CHK-265 or isotype mAb-treated mice (**Fig 4F**). However, beginning at 2 hpi, CHK-265 mAb-treated mice had reduced levels of viral RNA in the ipsilateral ankle compared to isotype mAb-treated mice, which continued through 8 hpi (**Fig 4F**). We next determined when active replication began in the ipsilateral foot of CHK-265 or isotype mAb-treated mice. During productive alphavirus infection, the subgenomic mRNA segment encoding the structural proteins (C-E3-E2-6K-E1) is transcribed preferentially compared to the genomic RNA [36]. Measurement of the ratio of subgenomic (E1 RNA) to genomic (nsP3 RNA) RNA by qRT-PCR at 4, 6, and 8 hpi showed that virus started replicating in both CHK-265 and isotype control mAb-treated mice at 8 hpi (**Fig 4G**). Since there was no difference detected in the onset of replication between the two groups, the reduced viral RNA levels before 8 h in the presence of CHK-265 suggests that fewer cells became infected. These results suggest that CHK-265 likely limits the number of cells initially infected.

### CHK-265 mAb treatment alters immune cell recruitment into skeletal muscle

**Fig 5:**
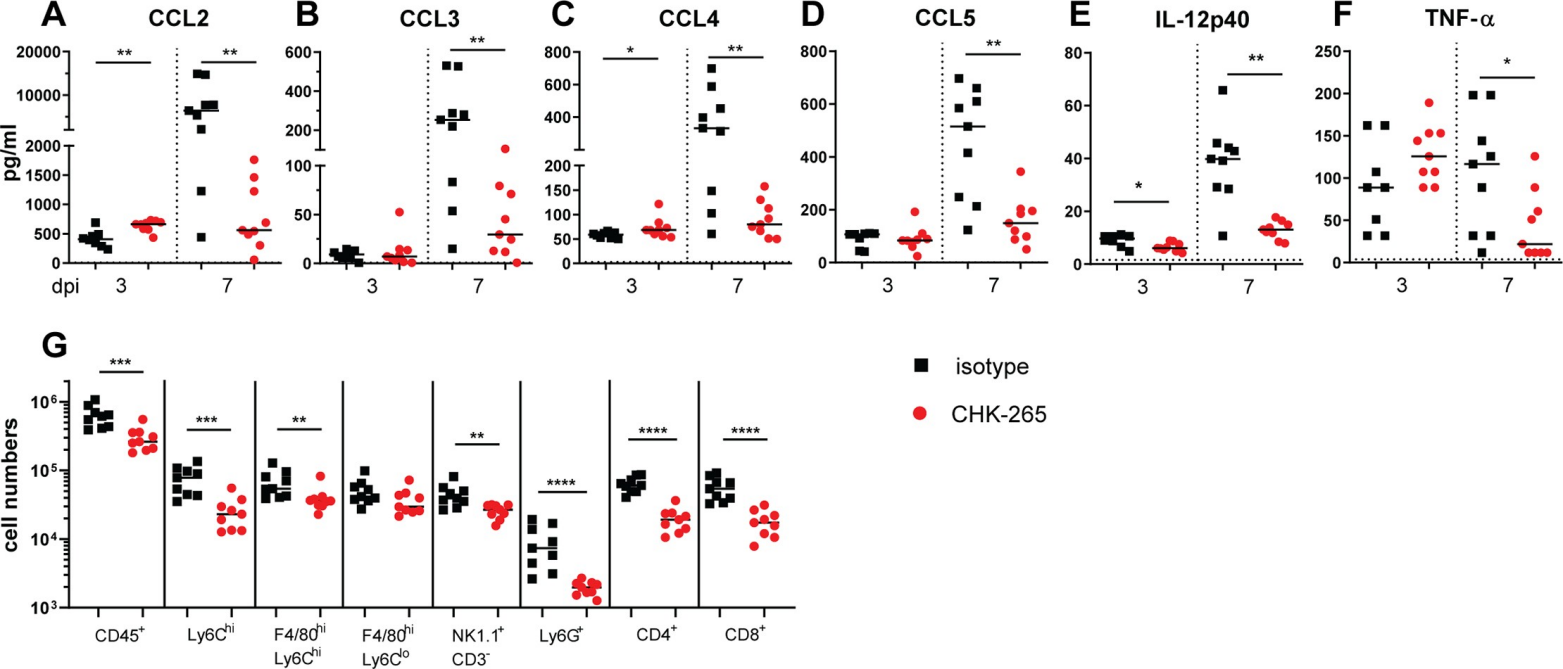
CHK-265 treatment results in reduced pro-inflammatory cytokines, chemokines, and immune cell infiltration in musculoskeletal tissue. Four-week-old WT male and female C57BL/6 mice were administered 100 μg of CHK-265 prior to infection with RRV. (**A-F**) Ipsilateral quadriceps muscles were harvested 3 or 7 dpi and analyzed for cytokine and chemokine levels. Bars indicate median values (n = 8–9 per group; two experiments; Mann-Whitney test; *P < 0.05, **P < 0.01). (**G**) At 7 dpi, ipsilateral quadriceps muscles were digested, single cell suspensions were stained for CD45^+^ leukocytes, Ly6C^hi^ monocytes, F4/80^hi^Ly6C^hi^ monocyte-derived macrophages, F4/80^hi^Ly6C^lo^ tissue resident macrophages, NK1.1^+^CD3^-^ NK cells, Ly6G^+^ neutrophils, CD4^+^ T cells, or CD8^+^ T cells, and analyzed by flow cytometry. Bars indicate median values (n = 9 per group; two experiments; Mann-Whitney test; **P < 0.01, ***P < 0.001, ****P < 0.0001).

Infiltration of immune cells, including neutrophils, monocytes, and CD4^+^ T cells, contribute to the pathogenesis of musculoskeletal disease during alphavirus infection in mice [37]. Since type I IFN signaling mediated CHK-265 reduction of RRV infection at the site of inoculation and type I IFNs can impact cellular activation and recruitment [38], we hypothesized that the early control of infection could reduce inflammation through modified immune cell recruitment into affected muscle tissue. To evaluate this idea, we treated mice with CHK-265 mAb prior to inoculation of RRV in the foot and analyzed levels of pro-inflammatory cytokines and chemokines at 3 and 7 dpi in the ipsilateral quadriceps muscles. At 3 dpi, there were minimal differences in pro-inflammatory cytokine or chemokine levels between CHK-265 and isotype control mAb treatment (**Fig 5A–5F**). However, by 7 dpi, the time of onset of disease in this model (**Fig 1C**), CHK-265 mAb treatment resulted in reduced levels of CCL2, CCL3, CCL4, CCL5, IL-12p40, and TNF-α compared to the isotype control mAb (**Fig 5A–5F**). Analysis of immune cell subsets at 7 dpi in the ipsilateral quadriceps muscles showed reduced numbers of CD45^+^ leukocytes, Ly6C^hi^ monocytes, F4/80^hi^Ly6C^hi^MHCII^+^ monocyte-derived macrophages, NK1.1^+^CD3^-^ NK cells, Ly6G^+^ neutrophils, and CD4^+^ and CD8^+^ T cells with CHK-265 administration compared to isotype mAb treatment (**Figs 5G and S3**). These results suggest that early control of the infection results in a reduced inflammatory response in musculoskeletal tissues, which likely limits clinical disease.

### Cross-reactive CHIKV immune polyclonal IgG reduces RRV-induced disease and selects for RRV variants in the E2 B domain

**Fig 6:**
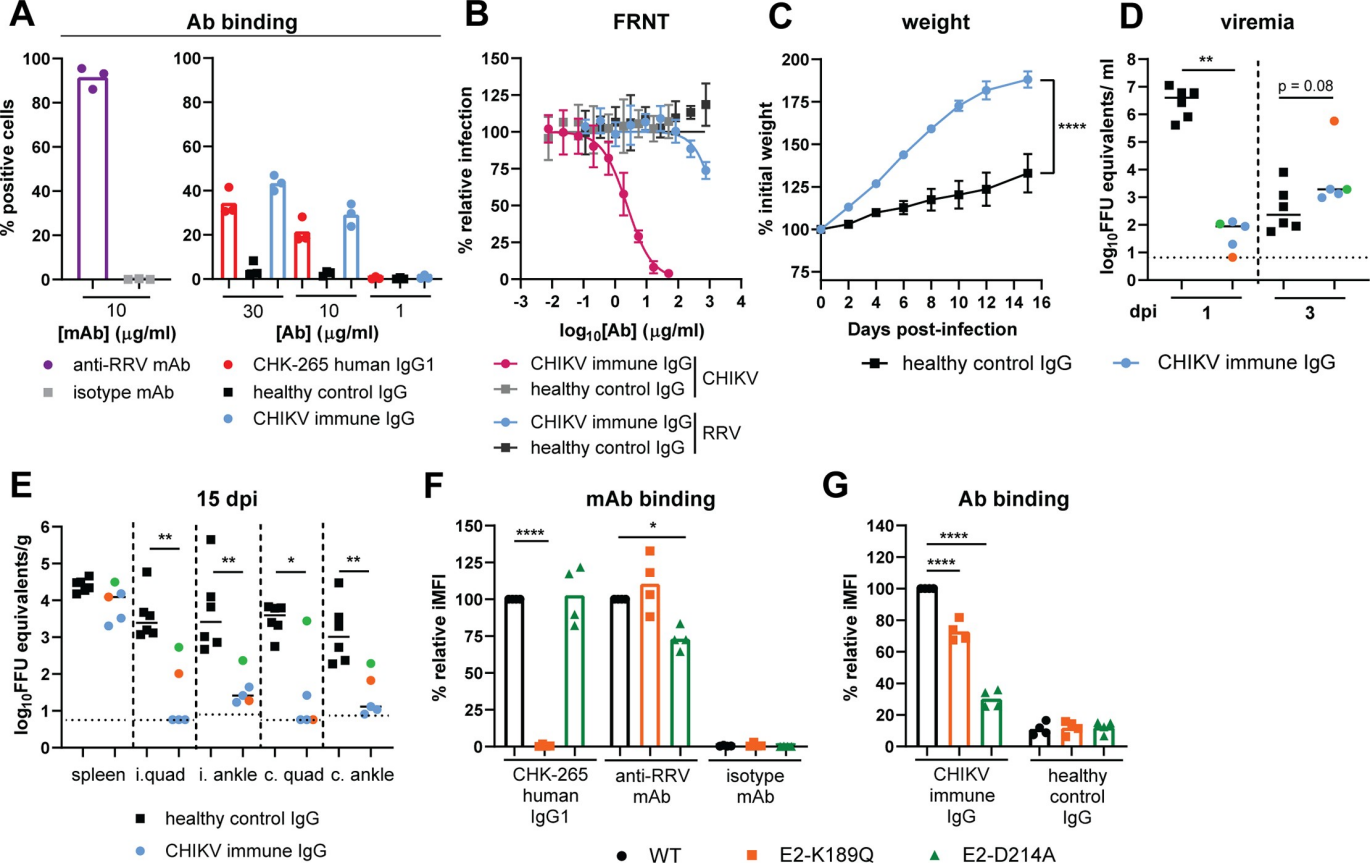
Cross-reactive CHIKV immune polyclonal IgG protects against RRV-induced disease despite emergence of RRV variants in the E2 B domain. (**A**) 293T cells were transfected with the RRV structural genes, stained using CHK-265 human IgG1, CHIKV immune IgG, or IgG isolated from healthy controls (healthy control IgG), and analyzed by flow cytometry. An anti-RRV mAb (RRV-130; 10 μg/ml) and human isotype control (WNV hE16; 10 μg/ml) were included as positive and negative controls, respectively. (**B**) Neutralization assay. Purified polyclonal CHIKV-immune IgG was preincubated with 10^2^ FFU of RRV or CHIKV-LR then added to Vero cells for 20 or 18 h, respectively. Viral foci were counted and compared to wells without mAb to determine relative infection. Purified IgG from healthy individuals (healthy control IgG) was included as a negative control. (**C**-**E**) Three-week-old male and female WT mice were administered 300 μg of purified human CHIKV immune IgG or healthy control IgG one day prior to infection with 10^3^ FFU RRV. (**C**) Weights were followed for 15 dpi. Graph shows mean ±SEM (n = 5–6 per group; two experiments; student’s t-test of AUC analysis: ****P < 0.0001). (**D**) Serum was collected 1 and 3 dpi and titered by qRT-PCR. (**E**) Viral RNA was quantified from spleen, ipsilateral (i.) and contralateral (c.) quadriceps muscles (quad), and ipsilateral and contralateral ankle at 15 dpi using qRT-PCR. (**D-E**) Bars represent the median (n = 5–6 per group; two experiments; Mann-Whitney test; *P < 0.05, **P < 0.01). Colored circles indicate CHIKV immune IgG treated mouse with identified RRV variant at 3 dpi: orange circle: E2-K189Q mutation; green circle: E2-D214A mutation. (**F**, **G**) 293T cells were transfected with the WT, E2-D214A, or E2-K189Q RRV structural genes, stained with (**F**) CHK-265 human IgG1, anti-RRV mAb [RRV-130; 0.04 μg/ml (EC_80_)], human isotype control (WNV hE16), (**G**) CHIKV immune IgG, or healthy control IgG at 30 μg/ml unless otherwise noted, and analyzed by flow cytometry. Relative integrated mean fluorescence intensity (iMFI) was calculated by multiplying the percent positive cells and the median fluorescence intensity for each RRV variant and comparing it to the RRV WT control. Bars indicate mean (four experiments performed in duplicate; One-way ANOVA with a Holm-Sidak’s post-test comparing RRV variants to WT; *P < 0.05, ****P < 0.0001). Ab: antibody.

We performed parallel experiments using cross-reactive polyclonal human antibodies to determine if the results we observed with CHK-265 also occur in the context of natural heterologous immunity. IgG was purified from an individual previously infected with CHIKV during the 2014 outbreak in Haiti [39]. The CHIKV immune IgG bound in a dose-dependent manner to RRV structural proteins on the cell surface at a level comparable to that of the chimeric human CHK-265 mAb, although considerably less than that of an RRV specific mAb (**Fig 6A, S4A and S4B Fig**). The CHIKV immune IgG efficiently neutralized CHIKV, but poorly neutralized RRV (**Fig 6B**).

To determine if CHIKV immune IgG protects mice from RRV-induced disease, 300 μg of the CHIKV immune IgG or IgG isolated from a healthy control human subject was administered one day prior to RRV infection. Mice receiving the CHIKV immune IgG had increased weight gain compared to healthy control IgG (**Fig 6C**). Viral RNA was analyzed in the serum at 1 and 3 dpi. Analogous to CHK-265, CHIKV immune IgG reduced viremia at 1 dpi but at 3 dpi, increased viremia was observed compared to the level of virus in animals treated with control IgG (**Fig 6D**). At 15 dpi, tissues were collected and analyzed for viral RNA. CHIKV immune IgG reduced viral RNA levels in the ipsilateral and contralateral quadriceps muscles and ankles compared to tissues from animals treated with the control IgG (**Fig 6E**).

To determine if RRV escape variants emerged in CHIKV immune IgG treated mice, we sequenced viral RNA isolated from the serum at 3 dpi. Sequencing revealed that one of the five viruses acquired an E2-K189Q mutation and a second had an E2-D214A mutation, whereas the remaining three viruses had no substitutions. Both of the mutations are located within the E2 B domain. The serum sample that contained the E2-K189Q had the highest titer at 3 dpi (**Fig 6D**; *orange circle*), and the mouse with the highest tissue titers at 15 dpi had the E2-D214A variant (**Fig 6E**; *green circle*). To determine the significance of these E2 variants, each mutation (E2-K189Q or E2-D214A) was introduced into an RRV structural gene cassette that was expressed in cells, and antibody binding to transfected cells was determined by flow cytometry. The E2-K189Q mutation abolished binding of the chimeric human CHK-265 to the RRV structural protein-expressing cells, but only slightly reduced binding of the CHIKV immune IgG (**S4C Fig, Fig 6F and 6G**). The E2-D214A mutation, however, reduced antibody binding of the CHIKV immune IgG and the anti-RRV mAb with minimal effect on CHK-265 binding (**Fig 6F and 6G**). Overall, these data indicate that a cross-reactive polyclonal anti-CHIKV response can reduce RRV disease *in vivo*, even with the rapid selection of antibody escape variants.

## Discussion

We examined whether moderately cross-neutralizing mouse monoclonal and human polyclonal antibodies generated in the context of CHIKV infection protect against RRV disease *in vivo*. Our goal was to model the effects of pre-existing alphavirus immunity derived from natural infection or vaccination on heterologous secondary infection. Indeed, there are several promising candidate CHIKV vaccines in clinical trials, including a virus-like particle, a measles-vectored virus, a live-attenuated virus, and mRNA-based platforms [40], which could generate cross-reactive immune responses. CHK-265 and CHIKV immune human IgG weakly neutralized the more distantly related RRV in cell culture, yet nonetheless reduced RRV-induced clinical disease in mice. Despite conferring clinical protection, RRV mutated rapidly to escape CHK-265 or CHIKV immune IgG neutralization or binding, which effectively shifted the kinetics of viral dissemination. Through a series of experiments in genetically-deficient mice and analysis of infection at early time points, we found that the early viral control at the inoculation site by CHK-265 depended on type I IFN signaling. This early difference in the response to RRV infection shaped the inflammatory response such that reduced levels of pro-inflammatory mediators and immune cell infiltrates accumulated in adjacent skeletal muscle tissues, which ultimately led to less disease severity.

CHK-265 mAb treatment did not eliminate RRV-induced musculoskeletal disease in mice but instead reduced its peak and duration. By extrapolation, pre-existing moderately cross-neutralizing antibody might mitigate but not prevent symptoms and disease during secondary alphavirus infection. This result contrasts with the phenotype expected from antibody-dependent enhancement (ADE) of viral infection by some cross-reactive antibodies [41]. Previous *in vitro* studies showed increased RRV replication in macrophages and monocytes in the presence of RRV-immune sera [42, 43]. Other studies in mice have suggested that suboptimal CHIKV humoral immunity can enhance homologous CHIKV disease. Administration of sub-neutralizing levels of mouse or human CHIKV immune sera or IgG resulted in increased foot swelling and viremia following CHIKV challenge [44], and aged mice vaccinated against CHIKV exhibited worse disease and viremia following CHIKV challenge compared to unvaccinated aged mice [45]. However, these studies did not confirm ADE *in vivo* through experiments with genetically modified mice (*e*.*g*. FcRγ^-/-^). Several factors may explain the differences between these studies and our results including homologous rather than heterologous virus challenge, relative antibody neutralization potency, the dose of antibody administered, and the age of the animals.

CHK-265 protected mice independently of Fc-FcγR interactions suggesting that virus neutralization was a primary mechanism of action. This finding contrasts with our recent study on the mode of optimal post-exposure therapeutic protection against homologous virus infection by mAbs (CHK-152 and CHK-166) [32]. Several methodological differences in that study likely contribute to the disparate outcomes: (a) the CHK-152 and CHK-166 mAbs were administered 3 dpi, which is after viral dissemination occurs; and (b) the mAbs were administered as a cocktail, which prevents viral escape [23]. RRV virions or RRV structural proteins on the surface of infected cells may have escaped binding of CHK-265 too rapidly for Fc effector functions to contribute to protection; indeed, Fc effector function-mediated protection by CHK-152 and CHK-166 requires infiltration of monocytes, which takes several days. Notwithstanding this point, and despite the emergence of neutralization escape viruses, the CHIKV immune IgG reduced RRV RNA levels through 15 dpi at local and distant sites compared to control IgG. While escape mutations reduced the binding of CHIKV immune IgG to cells expressing RRV structural proteins, the effect was partial, which could still allow for continued neutralization and Fc-dependent clearance of virus.

Alphaviruses can escape from antibody neutralization during mAb monotherapy *in vitro* and *in vivo* [16, 18, 24, 46–49]. Antibody escape variants were isolated from brain homogenates of CHIKV infected *Ifnar1*^*-/-*^ mice between 2 and 10 dpi [18, 48]. We identified RRV escape variants within 2 days of infection in both *Ifnar1*^*-/-*^ and WT mice in the serum and ankle tissues. Deep sequencing of the inoculating viral stock revealed that two of the five escape variants (E2-T219P and E2-D214A) pre-existed at relatively low frequencies (3.5% and 0.8%, respectively), even though the virus was propagated in the absence of any antibody. While the pre-existence of the E2-T219P variant virus in the inoculating stock likely contributed to the rapid escape from CHK-265, RRV variants at other critical contact residues (E2-D206E [4%] and E2-T210P [2.3%]), as defined by structural and mapping studies [29], also were present in the inoculating stock at similar frequencies but were not selected. The E2-T219P variant virus may have emerged *in vivo* because there was no negative effect of that mutation on viral fitness. Alignment of 295 RRV E2 sequences using the Virus Pathogen Resource (ViPR) database identified variants at E2-T219 [T→A (frequency, 4%)] from human isolates of RRV collected in American Samoa and the Cook Islands between 1979–1980 [50, 51]. Additionally, a variant at residue 214 [D→G (frequency, 0.3%)] was isolated in Australia from a human specimen [52]. Within the E2 sequence from the inoculating stock, the B domain had the highest frequency of nucleotide variation (21%) followed by the two arch domains (18–19%), the C domain (8%), and the A domain (6%). The B domain may tolerate mutations that facilitate immune evasion while still maintaining pathogenic potential, although the impact of these mutations on transmission and replication in mosquitoes remains unknown.

Similar to the experiments with CHK-265 mAb, RRV variants emerged with CHIKV immune IgG treatment by 3 dpi. Unlike CHK-265, where RRV variants were present in all mice, we identified RRV mutants in only two of the five serum samples. Low frequency RRV variants may have been present, but were below the threshold for detection by Sanger sequencing. The two RRV variants identified had mutations in the E2 B domain (E2-K189Q and E2-D214A) suggesting that this is a main epitope for cross-neutralizing anti-CHIKV antibodies against RRV even within a complex polyclonal antibody response. While the E2-D214A mutation was present in the inoculation stock at a low frequency (0.8%), the E2-K189Q mutation was not detected. At 3 dpi, the mouse with the highest viremia possessed the E2-K189Q mutation, whereas at 15 dpi, the mouse with the E2-D214A variant had the highest tissue viral load. We speculate that the E2-K189Q mutation reduces neutralization efficiency of the virion, resulting in enhanced viral load at early time points, but the CHIKV immune IgG maintains sufficient binding to forms of the envelope proteins on the cell surface that results in continued clearance of infected cells. Reciprocally, the E2-D214A variant had minimal impact on antibody neutralization, but could have a greater effect on antibody binding to the surface of infected cells, which facilitates clearance. For both RRV variants, a portion of the CHIKV immune IgG also might bind to other cross-reactive epitopes beyond the E2 B domain that aid in protection *in vivo*.

In the context of CHK-265 treatment, early events at the site of virus inoculation impacted local infection and clinical disease, whereas effects at distant sites were less prominent. Although CHK-265 treatment prevented initial viral dissemination, within 2 days, resistant RRV was detectable in distal tissues. When comparing the total virus burden in each tissue across all time points between CHK-265 and isotype mAb treatments, the serum, spleen, ipsilateral and contralateral quadriceps muscles, and contralateral ankle had similar amounts. However, the ipsilateral ankle had a 130-fold decrease in viral burden with CHK-265 administration, and this difference was Ifnar1-dependent. One explanation for these data is that the RRV neutralized by CHK-265, by virtue of its containing RNA PAMPs [*e*.*g*., non-2′-O methylation and secondary structural elements [53]], triggers pattern recognition receptors such as IFIT1 and toll-like receptors (TLRs) [54]. Consistent with this idea, TLR3 and TLR7 signaling has been implicated in the control of alphavirus infection [55, 56]. Indeed, at 8 hpi, animals treated with CHK-265 showed less RRV RNA, suggesting that the RNA is either cleared by phagocytic cells or degraded in the endosome. Alternatively, type I IFN responses could be suppressed in the isotype mAb-treated mice in the context of RRV replication due to virus-induced shut-off of host cell transcription and translation [57].

Type I IFN signaling in non-hematopoietic cells controls viral infection and foot swelling during CHIKV infections [58, 59] and can modulate immune cell recruitment and activation [38]. An early type I IFN response could activate certain immune cells subsets (*e*.*g*., such as NK cells [60]) that clear virus infection while limiting immune-mediated pathogenesis. In addition to the effects on joint-associated tissues, RRV infection also causes myositis [30]. CHK-265 treatment reduced pro-inflammatory cytokines and chemokines in the ipsilateral quadriceps muscle, which correlated with fewer infiltrating immune cells, including monocytes, neutrophils, and CD4^+^ and CD8^+^ T cells, all of which have been implicated in arthritogenic alphavirus disease pathogenesis [31, 61–63]. Thus, the early control at the site of infection could impact subsequent inflammation and disease pathogenesis.

One goal of vaccine studies is to induce broadly neutralizing antibodies that protect against multiple related viruses. With the global spread of CHIKV and the advanced development of two CHIKV vaccines, it is conceivable that the pre-existing immunity against CHIKV or other alphaviruses induced by natural infection or vaccination could become widespread. Our study shows that even moderately cross-neutralizing monoclonal or polyclonal antibodies developed against CHIKV could suppress heterologous RRV infection and reduce disease even if neutralization escape occurs. As these studies in mice show that cross-neutralizing antibodies can reduce heterologous alphavirus infection and disease severity, vaccine strategies that optimize such responses could have utility against multiple related alphaviruses.

## Materials and methods

### Monoclonal antibodies, cells, and viruses

CHK-265 and WNV E60 were purified from hybridoma supernatants as previously described [18]. CHK-265 chimeric mAbs with human Fc domains were generated recombinantly in ExpiCHO-S cells and tested free of endotoxins using the limulus ameocyte lysate test [32]. Vero and BHK21 cells were cultured in Dulbecco’s Modified Eagle Medium (DMEM) supplemented with 5% heat-inactivated fetal bovine serum (HI-FBS), HEPES pH 7.3, 100 U/L penicillin, and streptomycin at 37°C with 5% CO_2_. RRV T48 [RRV wild type (WT)] and RRV T184Q/A185S were produced from an infectious cDNA clone and passaged once in BHK21 cells [29, 35].

### Ethics statement

Experiments were performed in accordance with the recommendations in the *Guide for the Care and Use of Laboratory Animals* of the National Institutes of Health after approval by the Institutional Animal Care and Use Committee at the Washington University School of Medicine (assurance number: A3381-01). All infections in mice were performed under anesthesia with ketamine hydrochloride (80 mg/kg) and xylazine (15 mg/kg).

### Mouse studies

#### Clinical scoring experiments

Three-week-old WT male and female C57BL/6J were acquired from the Jackson Laboratory, and *FcR*γ^*-/-*^ mice were obtained from Taconic, backcrossed using speed congenic analysis onto a C57BL/6J background, and bred at the Washington University Animal Facility. CHK-265 or WNV E60 mAbs or purified human CHIKV immune IgG or healthy control IgG were administered to mice by intraperitoneal injection one day before subcutaneous inoculation in the left rear footpad with 10^3^ FFU of RRV in HBSS supplemented with 1% HI-FBS. Weights and clinical disease score were recorded daily or every other day. A clinical disease score was assigned based on hind paw grip strength, gait, and righting reflex [64]: 0, no disease signs; 1, mild defect in gripping of ipsilateral hind paw; 2, moderate bilateral defect in gripping of hind paws; 3, bilateral loss of gripping in hind paws, no change in gait; 4, bilateral loss of gripping in hind paws, moderate altered gait, difficulty righting self; 5, bilateral loss of gripping in hind paws, severely altered gait, unable to right self; 6, bilateral loss of gripping in hind paws, severely altered gait and dragging hind limb, unable to right self; 7, moribund.

#### Virological and immunological experiments

Four-week-old male and female WT C57BL/6J mice were purchased from the Jackson Laboratory. Congenic four-week-old male and female *FcR*γ^*-/-*^, *C1q*^*-/-*^, *Rag1*^*-/-*^, and *Ifnar1*^*-/-*^ C57BL/6J mice were obtained commercially (*FcR*γ^*-/-*^Taconic, and, C1q^*-/-*^, and *Rag1*^*-/-*^ Jackson Laboratories) or from colleagues (*Ifnar1*^*-/-*^, J. Sprent, Scripps) and bred at the Washington University Animal Facility. Mouse CHK-265, WNV E60 (mouse isotype control), chimeric human CHK-265 IgG1, chimeric human CHK-265 N297Q IgG1, or WNV hE16 (human isotype control) mAbs were administered to mice by intraperitoneal injection one day before subcutaneous inoculation in the left rear footpad with 10^3^ FFU of RRV, RRV T184Q/A185S, or RRV T219P in HBSS supplemented with 1% HI-FBS. Tissues were collected following extensive perfusion with PBS.

### Viral burden analysis

Perfused tissues were homogenized in DMEM supplemented with 2% HI-FBS, HEPES pH 7.3, and 100 U/ml penicillin and streptomycin using the MagNA Lyser (Roche). Clarified homogenate was titered by FFA using mouse RRV immune ascites fluid as the detecting antibody or by qRT-PCR. For qRT-PCR, RNA was isolated from tissue homogenates using RNeasy mini kit (Qiagen), and the quantity of RRV was determined using the TaqMan RNA to Ct one-step kit (Applied Biosystems) with nsp3 specific primers/probe (Forward: 5′-GTGTTCTCCGGAGGTAAAGATAG-3′, Reverse: 5′-TCGCGGCAATAGATGACTAC-3′, Probe: 5′-/56-FAM/ACCTGTTTA/ZEN/CCGCAATGGACACCA/3IABkFQ/-3′). RNA isolated from viral stocks was used as a standard curve to determine FFU equivalents. All viral titers were normalized to gram of tissue or ml of serum. The ratio of subgenomic RNA to genomic RNA also was determined. Viral RNA was quantified using nsp3 (genomic) and E1 (subgenomic) specific primers/probe (Forward: 5′-GCAGACACCATCCGGATTTA-3′, Reverse: 5′-GCTCTGACTGGATTGGTCTTTA-3′, Probe: 5′-/56-FAM/TGGCTGAAG/ZEN/GAGAAAGGATCTTCATTGA/3IABkFQ/-3′) with the TaqMan RNA to Ct one-step kit. RNA isolated from viral stocks was used as a standard curve to determine FFU equivalents.

### Flow cytometry

Perfused ipsilateral quadriceps muscles were minced and digested in RPMI supplemented with 10% HI-FBS, HEPES pH 7.3, 100 U/ml penicillin and streptomycin, collagenase (2.5 mg/ml; Sigma), and DNase I (30 μg/ml; Roche) in a total of 5 ml at 37°C for 1.5 h. Digested tissue was pressed through a 70 μm cell strainer, and resuspended in RPMI supplemented with 10% HI-FBS and penicillin and streptomycin. Cells were counted using precision count beads (BioLegend). Single cell suspensions were blocked for FcγR binding (BioLegend; clone S17011E) and stained with the following antibodies: CD3 BV421 (BioLegend; clone 145-2C11), CD4 FITC (BioLegend; clone RM4-5), CD8α APC (BioLegend; clone 53–6.3), NK1.1 PE (BioLegend; clone PK136), CD45 BUV395 (BD Biosciences; clone 30-F11), CD19 BV605 (BioLegend; clone 6D5), CD11b PerCP-Cy5.5 (BioLegend; clone M1/70), Ly6C Pacific Blue (BioLegend; clone HK1.4), Ly6G phycoerythrin (PE)-Cy7 (BioLegend; clone 1A8), MHC class II Alexa Fluor 700 (BioLegend; clone M5/114.15.2), Ly6B FITC (BioLegend; clone 7/4), and F4/80 BV650 (BioLegend; clone BM8). Viability was determined by exclusion of a fixable viability dye (eBiosciences; e506). Samples were run on a BD LSRFortessa X-20 flow cytometer and analyzed using FlowJo version 10 (FlowJo, LLC).

### Cytokine and chemokine analysis

Perfused ipsilateral quadriceps muscles were collected at 3 or 7 dpi and homogenized in PBS with 0.1% BSA. Cytokines and chemokines were analyzed using a Bio-Plex Pro Mouse Cytokine 23-Plex Assay kit (Bio-Rad) following the manufacturer’s instructions.

### Neutralization tests

Focus reduction neutralization tests (FRNT) were performed as described [29]. Briefly, serial dilutions of antibodies were incubated with 100 FFU of virus for 1 h at 37°C. Virus-antibody mixture was added to Vero cells for 1.5 h at 37°C followed by overlay of a MEM and 1% methylcellulose supplemented with 2% FBS, 100 U/L penicillin and streptomycin, and HEPES buffer. Cells were fixed at 20 to 22 hpi and stained with mouse RRV immune ascites fluid (ATCC) followed by an anti-mouse IgG conjugated to HRP. Foci were visualized using TrueBlue Peroxidase substrate (SeraCare) and counted using an ELISPOT reader (Cellular Technology Limited). EC_50_ values were determined by non-linear regression constraining the top of the graph to 100 and the bottom to 0.

### RRV escape variant viruses

CHK-265 mAb was administered to WT C57BL/6J mice by intraperitoneal injection one day before subcutaneous inoculation in the left rear footpad with 10^3^ FFU of RRV in HBSS supplemented with 1% HI-FBS. Serum was collected at 3 dpi or ipsilateral ankle tissue was collected at 2 dpi, and viral burden was titered by FFA. Virus (10^2^ FFU) derived from serum, ipsilateral ankle, or parental RRV stocks was preincubated with CHK-265 for 1 h at 37°C, added to Vero cells for 1.5 h at 37°C with 5% CO_2_, then overlayed with 1:1 methycellulose and MEM supplemented with 2% HI-FBS, HEPES pH 7.3, and penicillin and streptomycin. Cells were fixed with 1% PFA for 20 h later and developed with mouse RRV immune ascites fluid. Viral foci were counted and compared to wells with no mAb. Viral RNA was isolated from serum samples containing virus resistant to CHK-265 neutralization using the QIAamp viral RNA mini kit (Qiagen). cDNA was produced using SuperScript III First-Strand Synthesis kit (Invitrogen) and the structural proteins were sequenced using previously described primers [29].

### Mutagenesis of RRV infectious clone or RRV VLP construct

The mutation at position 219 (T → P) of the E2 gene of RRV was engineered into the pRR64 cDNA clone using Phusion high-fidelity DNA polymerase [New England Biolabs (NEB)] and mutagenesis primers (**S3 Table**). The cycling times were 98° for 30 sec, 18 cycles of 98°C for 30 sec, 50°C for 30 sec, 72°C for 7 min with a final extension at 72°C for 10 min. The parental plasmid was digested with DpnI (NEB) at 37°C for 5 h. The mutant clone was transformed into XL10-Gold ultracompetent cells (Agilent) and grown on LB agar supplemented with 100 μg/ml of carbenicillin. Virus was rescued as previously described [29]. Briefly, the plasmid was linearized by SacI (NEB), transcribed *in vitro* with SP6 polymerase (Invitrogen), and electroporated into BHK21 cells. The mutation was confirmed by sequencing viral RNA isolated from the viral stock as previously described [29].

The mutations at position 214 (D → A) and 184 (K → Q) of the E2 gene of RRV were engineered into a pcDNA3.1(+) mammalian cell expression plasmid expressing codon optimized RRV structural genes, capsid-E3-E2-6K-E1, using Phusion high fidelity DNA polymerase (NEB) and mutagenesis primers (**S3 Table**) with the same conditions described above. The parental plasmid was digested with DpnI (NEB) at 37°C for 3 h and transformed into One Shot Top10 chemically competent cells (Invitrogen) and grown on LB agar supplemented with 100 μg/ml of carbenicillin. The plasmids were sequenced to confirm mutations.

### Deep sequencing of RRV stock

RNA was isolated from RRV WT stock using QIAamp viral RNA mini kit (Qiagen), cDNA was produced using SuperScript III First-Strand Synthesis kit (Invitrogen), and the structural proteins were amplified as described [29]. RRV raw DNAseq data was generated on an Illumina MiSeq (2x250 bp) for a total of 1,114,020 reads (557,010 x 2). Raw data was processed using fastp 0.20.0 to remove low quality reads and trim adapter sequences. Processed data was aligned to the reference Ross River amplicon sequence (2.8 kb) using BBMap 38.51. From a total 1,099,922 processed reads, < 1% (1,314) did not map to the reference, whereas > 99% (1,098,608) reads mapped to the reference. Variants were called using LoFreq 2.1.3.1 with a P-Value cutoff of 0.01. All next-generation sequencing data set has been uploaded to the Sequence Read Archive (accession SRR11919531).

### Purification of human polyclonal IgG

CHIKV immune plasma was obtained from a donor in the convalescent phase of CHIKV infection as part of an IRB-approved study (Washington University Arthritis and Rheumatology Tissue Procurement Facility) [39]. IgG was isolated from CHIKV immune or healthy control plasma using Protein A/G spin columns (Thermo Scientific) following the manufacturer’s protocol. Purified IgG was dialyzed overnight in PBS in a 10 kDa Slide-A-Lyzer (Thermo Scientific), concentrated on a 50 kDa Amicon Ultra centrifugal filter (Millipore), and filter sterilized using a 0.22 μm PVDF filter (Millipore).

### Cell surface staining of RRV transfected cells

293T cells were reverse transfected with 1.25 μg per 1.25 x 10^6^ cells of the RRV WT, E2-D214A, or E2-K189Q structural gene expression construct using 3 μl of Lipofectamine 3000 and 3 μl of P3000 reagent following the manufacturer’s protocol. Twenty hours after transfection, cells were gently dislodged from the plate by pipetting. Cell suspensions were stained with CHK-265 human IgG1, CHIKV immune IgG, healthy control IgG, anti-RRV mAb (RRV-130), or human isotype control (WNV hE16) at indicated concentration in flow buffer (PBS + 1% FBS + 4mM EDTA) for 1 h at 4°C. Cells were washed and then stained with goat anti-human IgG (H+L) Alexa Fluor 647 (Invitrogen; 1:2000) for 30 min at 4°C. Cells were washed, fixed with 4% paraformaldehyde in PBS for 10 min at 4°C, then washed again to remove fixative. Cells were resuspended in flow buffer and ran on the MACSquant Analyzer 10 flow cytometer. Data was analyzed using FlowJo version 10. Integrated median fluorescence intensity (iMFI) was determined by multiplying the percent positive cells and the median fluorescence intensity of Alexa Fluor 647 for that population [65]. The background staining of the human polyclonal IgG to mock-transfected cells was subtracted from RRV transfected cells. We observed a low level of staining with the healthy control IgG in the RRV transfected cells that was not impacted by the mutations.

### Statistical analysis

Statistical significance was assigned with a *P* value of < 0.05 using GraphPad Prism version 8.2. The statistical test used for each graph is indicated in the Figure Legends. For animal weights and clinical scoring, area under the curve analysis was compared using an unpaired t-test or one-way ANOVA based on number of groups compared. Survival was analyzed using the log-rank test. For viral titers, the Mann-Whitney test or Kruskal-Wallis with Dunn’s post-test was used based on the number of groups compared. For cytokines, chemokines, and immune cell numbers, the Mann-Whitney test was used. For cell surface expressed RRV variants, a one-way ANOVA with a Holm-Sidak’s post-test comparing RRV variants to WT controls was used.

## Supporting information

S1 Fig CHK-265 shifts kinetics of RRV infection independent of activating FcγRs and C1q. Four-week-old male and female (**A**) FcRγ^-/-^ or (**B**) C1q^-/-^ C57BL/6 mice were administered 100 μg of CHK-265 one day prior to infection with 10^3^ FFU of RRV. Serum, spleen, ipsilateral (i.) and contralateral (c.) quadriceps muscles (quad), and ipsilateral and contralateral ankle were harvested 3 dpi and viral titers were determined by FFA [(A) n = 8 per group; two experiments; (B) n = 12–13 per group; three experiments].
(TIF)

S2 Fig Neutralization of virus derived from the ipsilateral ankle of WT mice. CHK-265 was preincubated with 10^2^ FFU of RRV derived from serum collected from CHK-265 or isotype-treated C57BL/6 WT mice at 3 dpi and then added to Vero cells for 20 h. Viral foci were counted and compared to wells without mAb to determine relative infection.
(TIF)

S3 Fig Gating scheme for infiltrating immune cells. WT male and female mice were administered 100 μg of CHK-265 one day prior to infection with 10^3^ FFU of RRV. Ipsilateral quadriceps muscles were harvested at 7 dpi and single cell suspensions were analyzed by flow cytometry. Gating scheme for (**A**) myeloid cells or **(B**) lymphocytes. The plots are representative of three experiments. Fixable viability dye: FVD.
(TIF)

S4 Fig Representative mAb staining of cell surface expressed RRV structural proteins. (**A-B**) 293T cells were transfected with the RRV structural genes (WT). (**A**) Cells were stained at indicated concentrations of CHK-265 human IgG1, CHIKV immune IgG, or IgG isolated from healthy controls (healthy control IgG), and analyzed by flow cytometry. (**B**) An anti-RRV mAb (RRV-130; 10 μg/ml) and human isotype control (WNV hE16; 10 μg/ml) were included as positive and negative controls, respectively. (**C**) 293T cells were transfected with the WT, E2-D214A, or E2-K189Q RRV structural genes, stained using CHK-265 human IgG1, CHIKV immune IgG, or healthy control IgG, and analyzed by flow cytometry. An anti-RRV mAb [RRV-130; 0.04 μg/ml (EC_80_)] and human isotype control (WNV hE16; 10 μg/ml) were included as positive and negative controls, respectively.
(TIF)

S1 Table Frequency of RRV escape mutations in viral stock. (PDF)

S2 Table Frequency of RRV variants in inoculating stock at critical contact residues identified by cryo-EM and alanine scanning mutagenesis. (PDF)

S3 Table Mutagenesis Primers. (PDF)

S1 Data Raw data files. Excel file containing the raw numerical values used to generate results, including Figs 1A–1F, 2A–2H, 2J–2O, 3A, 3E–3G, 4A-4G, 5A–5G, 6A–6G, S1A and S1B, S2. Each figure is separated into individual tabs.
(XLSX)
