# Supplementary figures and images for: A cross-reactive antibody protects against Ross River virus musculoskeletal disease despite rapid neutralization escape in mice
Source: PLoS Pathog. 2020 Aug 6;16(8):e1008743. doi: 10.1371/journal.ppat.1008743 (PMC7433899; doi:10.1371/journal.ppat.1008743)

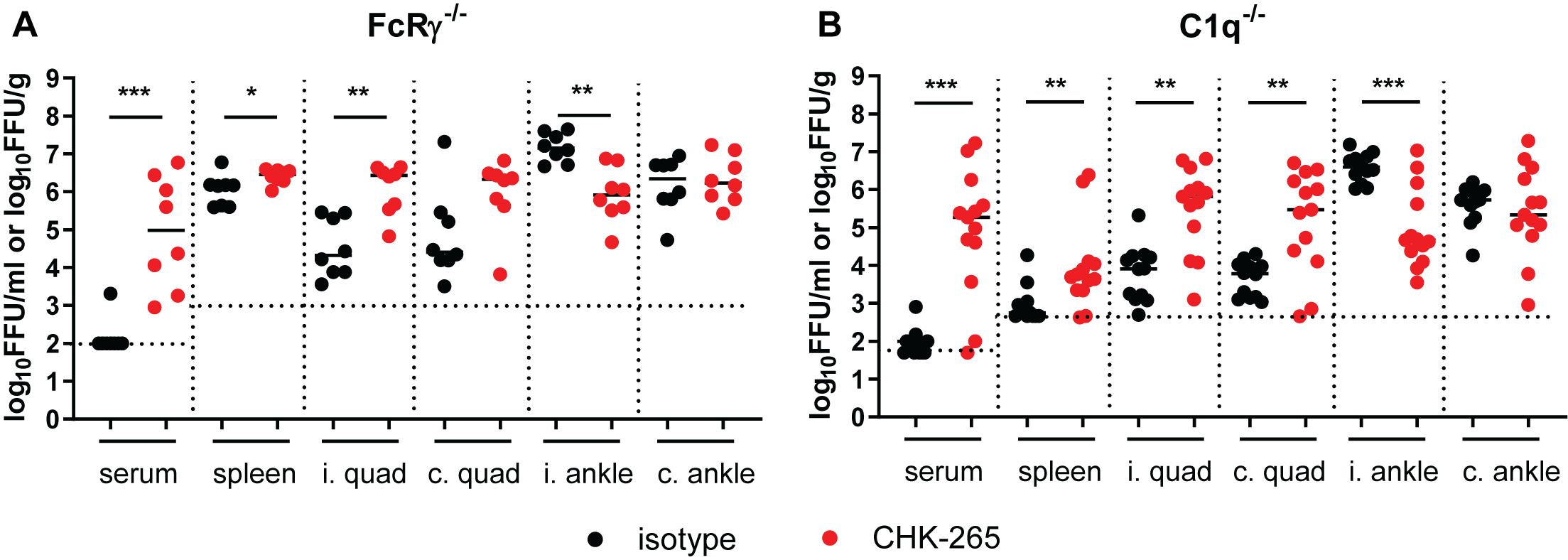

Supplement: S1 Fig — Four-week-old male and female (A) FcRγ-/- or (B) C1q-/- C57BL/6 mice were administered 100 μg of CHK-265 one day prior to infection with 103 FFU of RRV. Serum, spleen, ipsilateral (i.) and contralateral (c.) quadriceps muscles (quad), and ipsilateral and contralateral ankle were harvested 3 dpi and viral titers were determined by FFA [(A) n = 8 per group; two experiments; (B) n = 12–13 per group; three experiments]. (TIF) [file ppat.1008743.s001.tif]

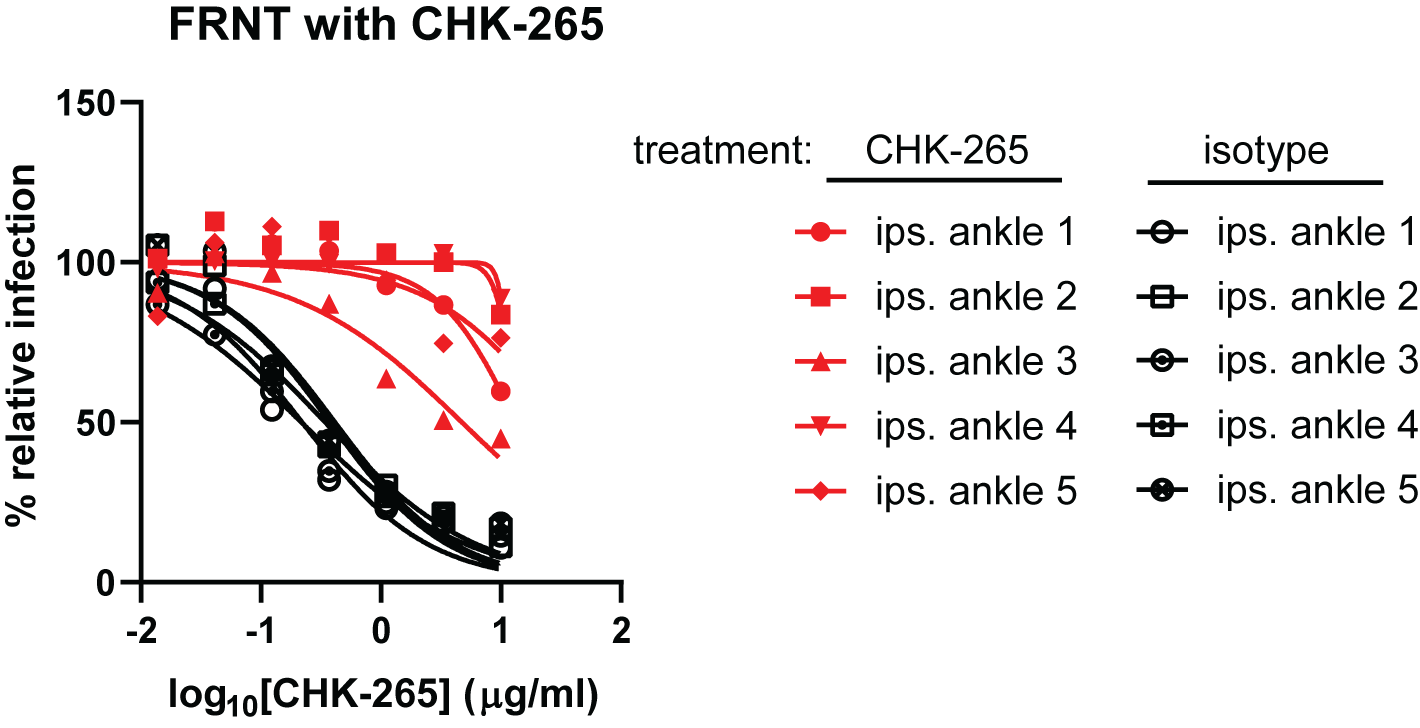

Supplement: S2 Fig — CHK-265 was preincubated with 102 FFU of RRV derived from serum collected from CHK-265 or isotype-treated C57BL/6 WT mice at 3 dpi and then added to Vero cells for 20 h. Viral foci were counted and compared to wells without mAb to determine relative infection. (TIF) [file ppat.1008743.s002.tif]

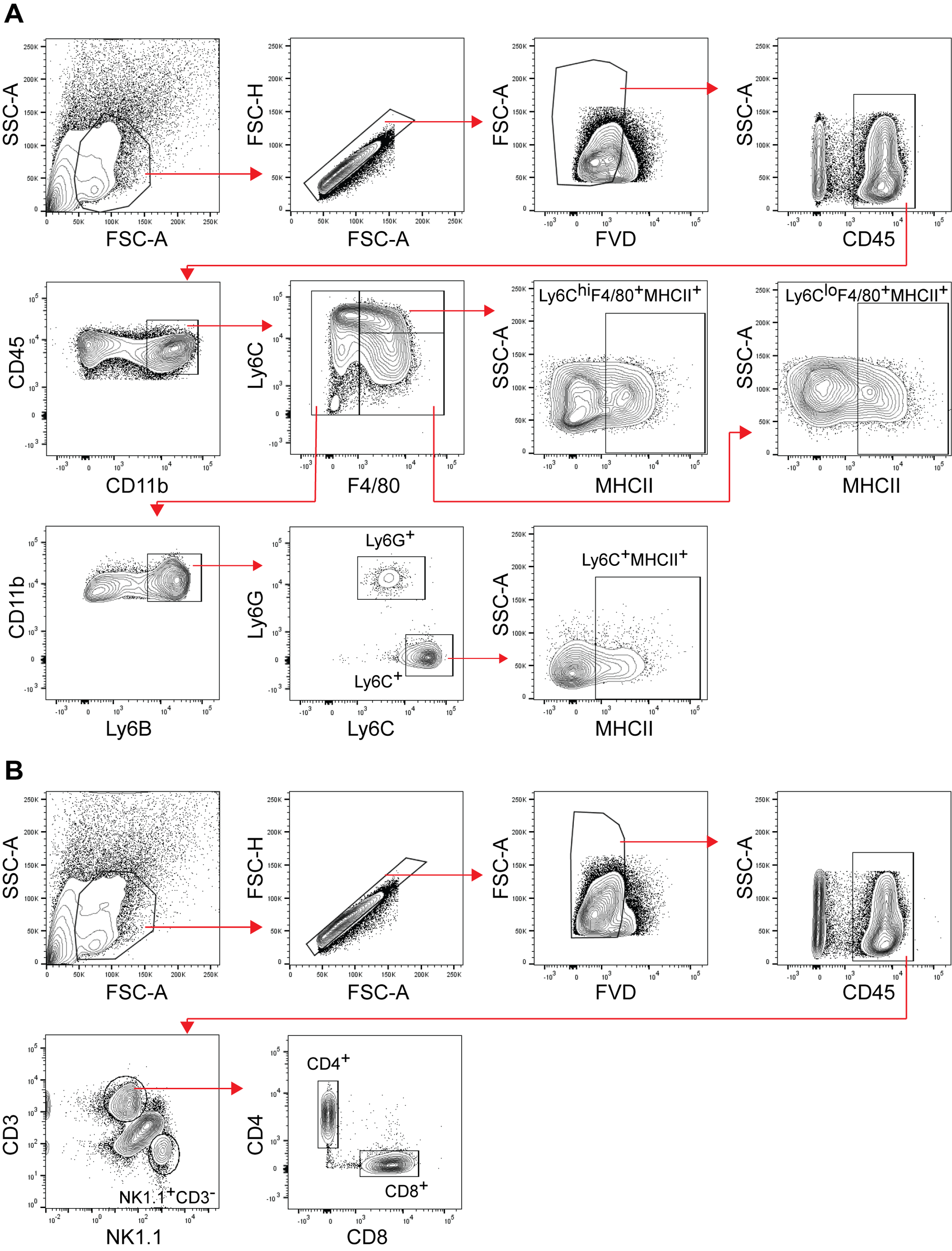

Supplement: S3 Fig — WT male and female mice were administered 100 μg of CHK-265 one day prior to infection with 103 FFU of RRV. Ipsilateral quadriceps muscles were harvested at 7 dpi and single cell suspensions were analyzed by flow cytometry. Gating scheme for (A) myeloid cells or (B) lymphocytes. The plots are representative of three experiments. Fixable viability dye: FVD. (TIF) [file ppat.1008743.s003.tif]

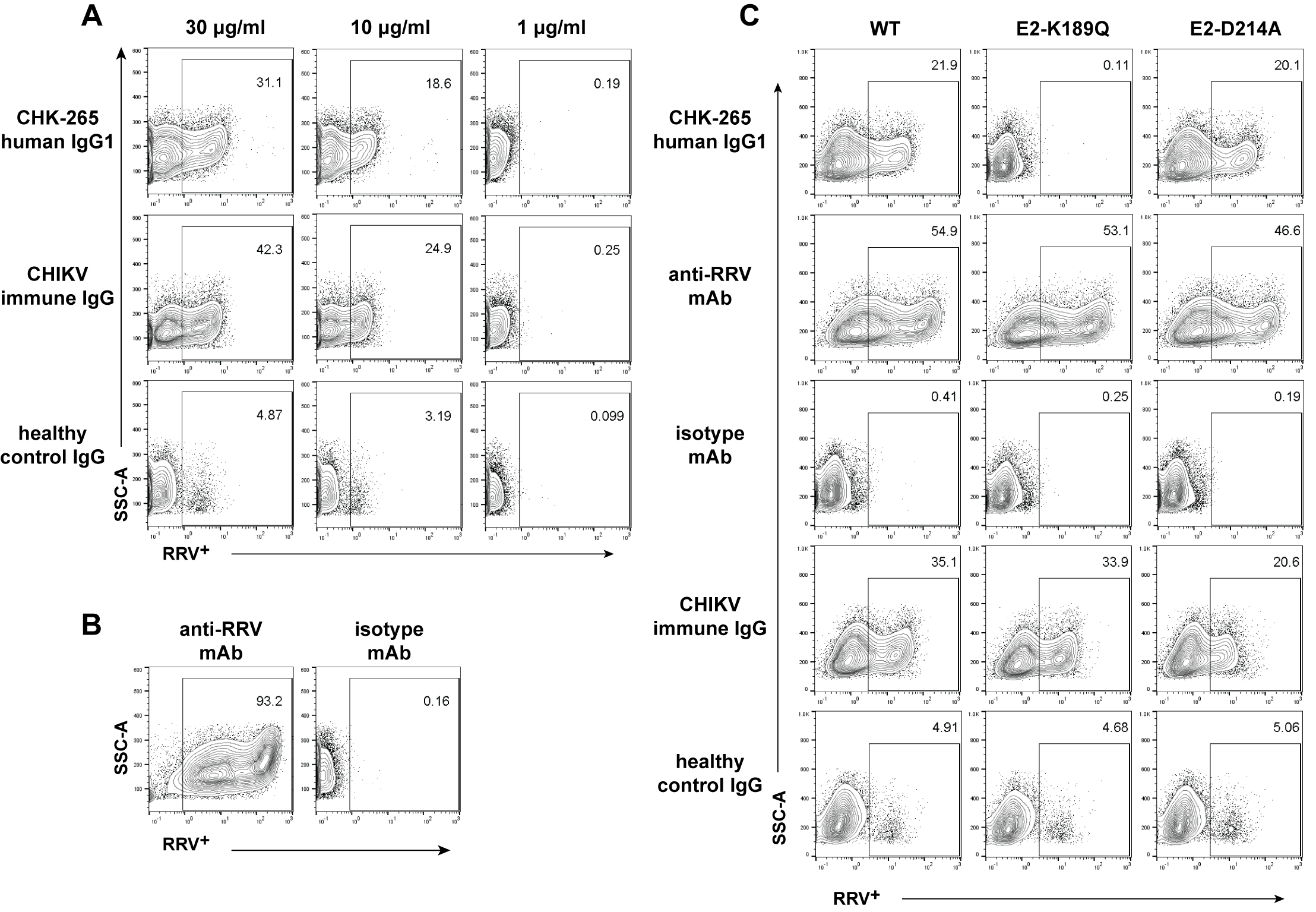

Supplement: S4 Fig — (A-B) 293T cells were transfected with the RRV structural genes (WT). (A) Cells were stained at indicated concentrations of CHK-265 human IgG1, CHIKV immune IgG, or IgG isolated from healthy controls (healthy control IgG), and analyzed by flow cytometry. (B) An anti-RRV mAb (RRV-130; 10 μg/ml) and human isotype control (WNV hE16; 10 μg/ml) were included as positive and negative controls, respectively. (C) 293T cells were transfected with the WT, E2-D214A, or E2-K189Q RRV structural genes, stained using CHK-265 human IgG1, CHIKV immune IgG, or healthy control IgG, and analyzed by flow cytometry. An anti-RRV mAb [RRV-130; 0.04 μg/ml (EC80)] and human isotype control (WNV hE16; 10 μg/ml) were included as positive and negative controls, respectively. (TIF) [file ppat.1008743.s004.tif]
