# Supplementary material for: A cross-reactive antibody protects against Ross River virus musculoskeletal disease despite rapid neutralization escape in mice
Source: PLoS Pathog. 2020 Aug 6;16(8):e1008743. doi: 10.1371/journal.ppat.1008743 (PMC7433899; doi:10.1371/journal.ppat.1008743)
Supplement: S1 Table — (PDF) [file ppat.1008743.s005.pdf]

Table S1. Frequency of RRV escape mutations in viral stock

Residue	E2 nucleotide substitution	amino acid change	%*	E2 nucleotide substitution	amino acid change	%*
**Q183**	A560U	H	0			
**T219**	A655C	P	3.5	C656A	K	1.0

*Percentage of nucleotides with substitution
