# Supplementary material for: A cross-reactive antibody protects against Ross River virus musculoskeletal disease despite rapid neutralization escape in mice
Source: PLoS Pathog. 2020 Aug 6;16(8):e1008743. doi: 10.1371/journal.ppat.1008743 (PMC7433899; doi:10.1371/journal.ppat.1008743)
Supplement: S2 Table — (PDF) [file ppat.1008743.s006.pdf]

Table S2. Frequency of RRV variants in inoculating stock at critical contact residues identified by cryo-EM or alanine scanning mutagenesis

	Residue	E2 nucleotide substitution	amino acid change	%*	E2 nucleotide substitution	amino acid change	%*
**Cryo-EM**	G72	N/A					
	H73	N/A					
	D74	N/A					
	T184	N/A					
	A185	N/A					
	N187	N/A					
	K189	N/A					
	**R205**	U615G	silent	2.4			
	**D206**	C618A	E	2.3			
	**D214**	A641C	A	0.8			
	**K215**	G645A	K	1.1			
	T216	N/A					
	**N218**	A653C	T	0.6	C654A	K	0.9
	**T219**	A655C	P	3.5	C656A	K	1.0
**Alanine scanning mutagenesis**	T184	N/A					
	A185	N/A					
	I190	N/A					
	I197	N/A					
	**Y199**	A596C	S	0.9	C597A	stop	1.0
	G209	N/A					
	**T210**	A628C	P	4.0			
	**S212**	U636G	R	0.7			
	**I217**	U650C	F	0.9			

*Percentage of nucleotides with substitution
