# Supplementary material for: A cross-reactive antibody protects against Ross River virus musculoskeletal disease despite rapid neutralization escape in mice
Source: PLoS Pathog. 2020 Aug 6;16(8):e1008743. doi: 10.1371/journal.ppat.1008743 (PMC7433899; doi:10.1371/journal.ppat.1008743)
Supplement: S3 Table — (PDF) [file ppat.1008743.s007.pdf]

**Table S3. Mutagenesis Primers**

| Mutation | Primers |
| --- | --- |
| E2- T219P | 5′- TACTGACAAGACCATCAACCCATGCAAGATTGACCAATG -3′ |
| | 5′- CATTGGTCAATCTTGCATGGGTTGATGGTCTTGTCAGTA -3′ |
| E2- D214A | 5′- AGGTATTGATTGTCTTAGCGGTGGATGTGGTGCC -3′ |
| | 5′- GGCACCACATCCACCGCTAAGACAATCAATACCT -3′ |
| E2- K189Q | 5′- CGCCGGCAACGTGCAGATCACAGCCGG -3′ |
| | 5′- CCGGCTGTGATCTGCACGTTGCCGGCG -3′ |
